# Genome-Wide Identification of β-D-Xylosidase Gene Family in Potato and Functional Analysis Under Alkaline Stress

**DOI:** 10.3390/plants14243790

**Published:** 2025-12-12

**Authors:** Shuangshuang Zheng, Xia Zhang, Caicai Lin, Peiyan Guan, Lu Liu, Mengyu Su, Qingshuai Chen, Ru Yu, Lingling Jiang, Ke Yao, Linshuang Hu

**Affiliations:** 1 Institute of Biophysics, Dezhou University, Dezhou 253023, China; zhangxia@dzu.edu.cn (X.Z.); lcc@dzu.edu.cn (C.L.); smy@dzu.edu.cn (M.S.); qingschen_dzu@dzu.edu.cn (Q.C.); angelyuru@163.com (R.Y.); jll@dzu.edu.cn (L.J.); 17794539779@163.com (K.Y.); 2 College of Life Science, Dezhou University, Dezhou 253023, China; peiyanguan@dzu.edu.cn (P.G.); liulufh2025@163.com (L.L.)

**Keywords:** *Solanum tuberosum*, genes, β-D-xylosidase (BXL), expression pattern, alkali

## Abstract

β-D-xylosidase (BXL) is a key enzyme involved in xylan degradation and plays crucial roles in plant development and stress responses. However, its functional roles in potato (*Solanum tuberosum*) remain poorly understood. In this study, we performed a genome-wide identification of the *StBXL* gene family and identified eight *StBXL* genes distributed across five chromosomes. A phylogenetic analysis classified these genes into four groups. Conserved motif and domain analyses indicated functional conservation among StBXL proteins. Analyses of cis-acting elements in the promoters and expression profiles of *StBXL* genes in various plant tissues under different stress treatments revealed their spatiotemporal expression patterns, as well as potential roles in phytohormone signaling and stress responses. Alkali stress significantly inhibited the expression of *StBXL4* and *StBXL5.* The overexpression of *StBXL4* enhanced the sensitivity of potato seedlings to alkali stress, whereas the overexpression of *StBXL5* showed no significant phenotypic differences under the same conditions. These results suggest that *StBXL4* acts as a negative regulator of the alkali stress response in potato. This study fills the research gap regarding the potato *StBXL* gene family and provides valuable insights for the molecular breeding of alkali-tolerant potato varieties.

## 1. Introduction

Plant growth and development are influenced not only by genetic factors but also by external environmental factors [1]. These environmental factors include abiotic stresses (e.g., high temperature, low temperature, drought, and salinity) as well as biotic stresses (e.g., pathogen infection, human activity, and attacks by animals) [2]. As the first line of defense for plants to perceive external signals, the cell wall is mainly composed of cellulose, hemicellulose, and pectin [3]. In addition to providing structural support and protection, the cell wall can perceive environmental changes, transmit signals to the interior of cells step by step, and trigger plant responses [4,5]. Hemicellulose accounts for 30–35% of the dry weight of plant cell walls, with xylan and mannan as the main components. During cell wall maturation, xylan is hydrolyzed by a series of enzymes, including endo-β-1,4-xylanase, β-D-xylosidase, α-L-arabinofuranosidase, acetyl xylan esterase, and phenol esterase [6].

β-D-xylosidase (BXL) is a critical member of the xylan hydrolase system that can synergistically hydrolyze xylan with xylanase [7]. The *BXL* gene encoding β-D-xylosidase is widely present in bacteria [8], fungi [9], and plants [10]. Members of the *BXL* gene family have been identified in plants, such as *Arabidopsis* [11], poplar (*Populus trichocarpa*) [12], barley (*Hordeum vulgare*) [13], and soybean (*Glycine max*) [14]. Typically, BXL proteins consist of an N-terminal catalytic domain (Glyco_hydro_3, PF00933), a C-terminal catalytic domain (Glyco_hydro_3_C, PF01915), and a fibronectin type III domain (Fn3-like, PF14310), whose functions remain incompletely elucidated. The Glyco_hydro_3 domain contains WGR (tryptophan–glycine–arginine) and KH (lysine–histidine) motifs that are related to substrate binding [10]. Additionally, BXLs generally possess two catalytic amino acid active sites: Asp (a nucleophilic catalytic residue) and Glu (an acid–base catalytic residue) [15].

In Arabidopsis, *AtBXL1* is specifically expressed in the stems, and *atbxl1* mutants exhibit shorter stems, smaller siliques, and a reduced seed yield [11]. *AtBXL4* plays a crucial role in systemic acquired resistance [16]. In the Japanese pear (*Pyrus pyrifolia* Nakai), *JPRXYL* is highly expressed in unripe fruits, ripe fruits, and senescent leaves, implying its involvement in the regulation of plant senescence [17]. In strawberry, the promoter region of *FaXyl1* contains multiple hormone-responsive elements, including those responsive to abscisic acid (ABA), indole-3-acetic acid (IAA), and gibberellin (GA). The exogenous application of GA_3_ and 1-naphthaleneacetic acid (NAA) downregulates *FaXyl1* expression, which is accompanied by reduced β-glucosidase activity [18]. In poplar, *PtBXL4*, *PtBXL5*, and *PtBXL9* are induced by ammonium and nitrate treatments, and nitrogen indirectly regulates their expression to control secondary cell wall formation [12]. Although *BXLs* have been identified in diverse plants species, the functional characterization of these genes remains relatively limited.

Potato (*Solanum tuberosum*) is the third most important food crop globally, characterized by low water consumption, high photosynthetic efficiency, strong environmental adaptability, and high yield potential [19,20,21,22]. As a crucial raw material for food and industrial processing, it plays a pivotal role in safeguarding national food security [23,24,25,26]. Soil salinization and alkalization are widespread abiotic stresses that become major limiting factors for global crop production [27,28,29]. Salt stress is a primary abiotic stress affecting potato growth [30,31,32]. Under salt stress, potato plants undergo significant physiological and morphological alterations, leading to a reduced tuber yield and quality [33,34]. Compared with neutral salt stress, alkali stress not only involves high salt concentrations but also high pH levels, causing combined damage from osmotic stress, ion toxicity, and oxidative stress [35,36,37,38,39]. The completion of potato genome sequencing has provided essential resources for the genome-wide analysis of the *StBXL* gene family [40]. However, to date, no comprehensive studies have been performed on the functions of *StBXL* genes and their regulatory mechanisms under alkaline stress in potato.

To characterize the *StBXL* genes in potato and investigate their molecular function, we performed a genome-wide identification and characterization of the *BXL* gene family in potato. In this study, we identified eight *StBXL* genes from the potato genome, investigated their distribution, and systematically analyzed their structure, chromosomal location, conserved motifs, cis-acting regulatory elements in their promoter regions, phylogenetic classification, and functional protein interaction networks. Additionally, we studied the expression of *StBXLs* in Xisen 6 under alkaline stress. We found that the overexpression of *StBXL4* significantly reduced the alkaline stress resistance of potato seedlings, indicating that *StBXL4* played a negative regulatory role in the alkaline stress response of potato. This study helps clarify the functions of *StBXL* genes and provides a theoretical and practical foundation for their application in molecular crop improvement.

## 2. Results

### 2.1. Identification and Characterization of StBXLs

In this study, eight *StBXL* genes were identified in the potato genome, named *StBXL1* to *StBXL8* according to their chromosomal locations. Coding and protein sequences are listed in Table S1. The detailed information on all members was analyzed, including the protein length, molecular weight, theoretical isoelectric point (pI), instability index, and subcellular localization prediction (Table 1). The number of amino acids of the StBXL proteins ranged from 773 amino acids (StBXL8) to 836 amino acids (StBXL7). The molecular weights of StBXL proteins ranged from 83.82 kDa (StBXL1) to 91.20 kDa (StBXL7). The predicted pI ranged from 6.10 (StBXL3) to 9.09 (StBXL6), indicating that the proteins differ in charge. The instability indices ranged from 28.24 to 39.42, indicating that all proteins were stable. Three StBXLs were localized to the Chloroplast (StBXL1, StBXL5, and StBXL8), StBXL3 and StBXL6 were localized to the plasma membrane, StBXL2 was localized to the endoplasmic reticulum, StBXL4 was localized to the extracellular region, and StBXL7 was localized to the vacuole. The different subcellular localizations of StBXL proteins may indicate that they have different functions under various conditions.

### 2.2. Chromosome Localization and Duplication of StBXLs

**Figure 1:**
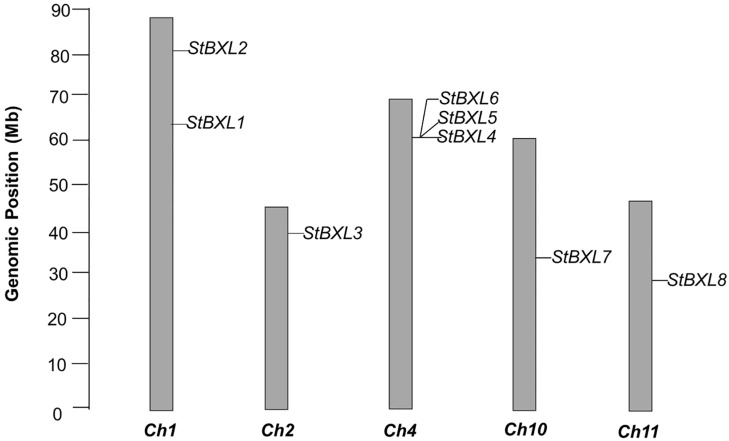
Genomic distribution of *StBXL* genes on potato chromosomes. Chromosome numbers are below. Scale bar on the left indicates chromosome length.

To characterize the distribution of *StBXs* in the potato genome, their chromosomal locations were determined using SpudDB data. The eight *StBXLs* were unevenly mapped to five chromosomes: two on chromosome 1, one on chromosome 2, three on chromosome 4, and one each on chromosomes 10 and 11 (Figure 1).

Tandem and segmental duplications contributed to the formation of the gene family. *StBXL4*, *StBXL5*, and *StBXL6* constitute a cluster of tandemly duplicated genes on chromosome 4 (Table S2). The Multiple Collinearity Scan toolkit (MCScanX) was used to analyze the synteny blocks among potato chromosomes to clarify the evolution of potato *StBXLs*, which revealed no segmentally duplicated *StBXL* genes in the potato genome. This finding indicated that *StBXL* genes are evolutionarily conserved.

### 2.3. Phylogenetic and Synteny Analysis of BXL in Different Plant Species

**Figure 2:**
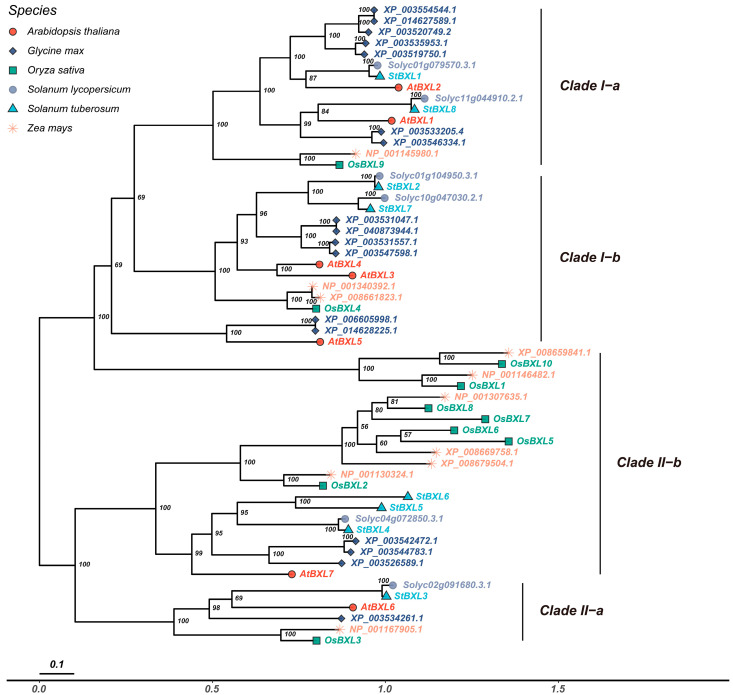
Phylogenetic analyses of StBXL proteins. A phylogenetic tree was constructed for the BXL protein sequences of potato (*Solanum tuberosum*), maize (*Zea mays*), Arabidopsis (*Arabidopsis thaliana*), rice (*Oryza sativa*), soybean (*Glycine max*), and tomato (*Solanum lycopersicum*), and it was divided into four subgroups. The values on the branches of the phylogenetic tree were the bootstrap values, which represent percentages.

To clarify the taxonomic and evolutionary relations of the *StBXL* gene family, a neighbor-joining phylogenetic tree was constructed using 58 protein sequences from Arabidopsis (*Arabidopsis thaliana*), tomato (*Solanum lycopersicum*), soybean (*Glycine max*), maize (*Zea mays*), and rice (*Oryza sativa*) (Figure 2). The results showed that StBXL proteins were categorized into four subfamilies, and proteins in the same subgroup were more closely related. StBXL1 and StBXL8 clustered in clade I-a, whereas StBXL2 and StBXL7 clustered in clade I-b. These two clades also contained BXL proteins from Arabidopsis, soybean, and tomato, suggesting that these proteins are evolutionarily close. StBXL4, StBXL5, and StBXL6 clustered in clade II-b, whereas StBXL3 clustered in clade II-a. StBXL belongs to a different major branch from the BXL proteins of rice and maize, suggesting that the BXL proteins had a undergone evolutionary divergence in different plants, whereas the closest affinity to tomato suggested that BXLs were functionally similar in potato and tomato. In conclusion, StBXL proteins in the same clade had closer affinities and might be functionally conserved, and StBXL proteins in different branches might have large functional differences, which were also related to their subcellular localization.

**Figure 3:**
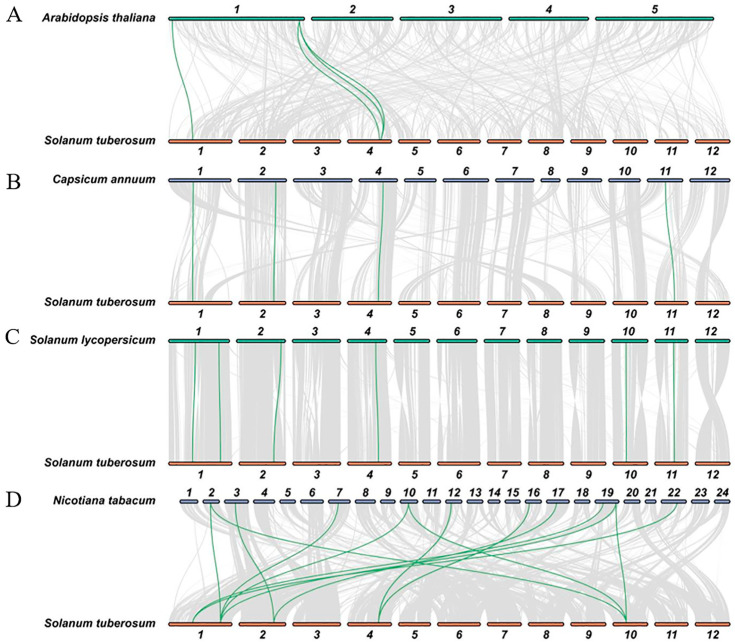
Synteny analysis of *StBXL* genes between potato and four other plant species. Gray lines at bottom indicate collinear blocks with potato and other plant genomes. Green lines indicate *BXL* gene pairs. Results of synteny analyses between potato and Arabidopsis (**A**), pepper (*Capsicum annuum*) (**B**), tomato (**C**), and tobacco (*Nicotiana tabacum*) (**D**).

We performed a collinearity analysis of *StBXL* genes in different plant species to study the molecular evolutionary relations of *BXL* genes. The numbers of conserved *BXL* gene pairs between potato and Arabidopsis, tomato, pepper, and tobacco were four, six, four, and twenty-seven, respectively (Figure 3).

### 2.4. Gene Structure, Conserved Domain, and Motif Analyses of StBXL

**Figure 4:**
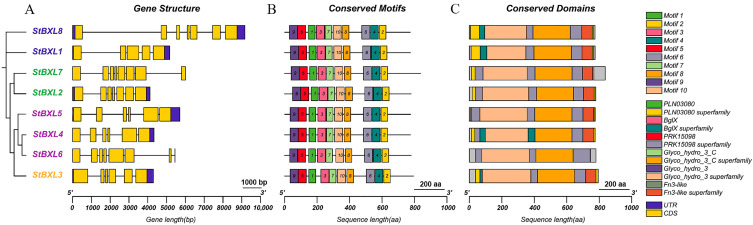
Gene structures of *StBXL* genes and conserved motifs and domains of StBXL proteins. (**A**) Structural analysis of *StBXL* genes. Blue rectangles, yellow rectangles, and black lines represent UTR, CDS, and introns, respectively. Scale bar = 1000 bp. (**B**) Conserved motif analysis of StBXL proteins. Top 10 motifs identified from potato protein obtained by MEME analysis through TBtools-II. Scale bar = 200 aa. (**C**) Conserved domain analysis of StBXL proteins. Scale bar = 200 aa.

To elucidate the diversity and similarities among members of the *StBXL* gene family, the gene structures, conserved domains, and motifs of StBXL proteins were systematically analyzed. The gene structure analysis revealed that *StBXL7* contained eight exons; *StBXL1*, *StBXL2*, and *StBXL6* each had seven exons; *StBXL3*, *StBXL4*, and *StBXL5* had six exons; and *StBXL8* had five exons. All *StBXL* genes contained 5′ and 3′ untranslated regions (UTRs) except *StBXL6* and *StBXL7*, which lacked both 5′ and 3′ UTRs, and *StBXL4*, which lacked the 5′ UTR (Figure 4A). *StBXL* genes clustered in the same clade exhibited conserved gene structural features. StBXL proteins’ motif distribution was investigated, and 10 putative conserved motifs were identified (Figure 4B), the details of which are illustrated in Figure S1. All StBXL proteins contained the Glyco_hydro_3, Glyco_hydro_3_C, and Fn3-like domains except for StBXL6 (Figure 4C). These results demonstrate the conservation of the StBXL proteins’ structure.

### 2.5. Analysis of Cis-Elements in StBXL Promoters

**Figure 5:**
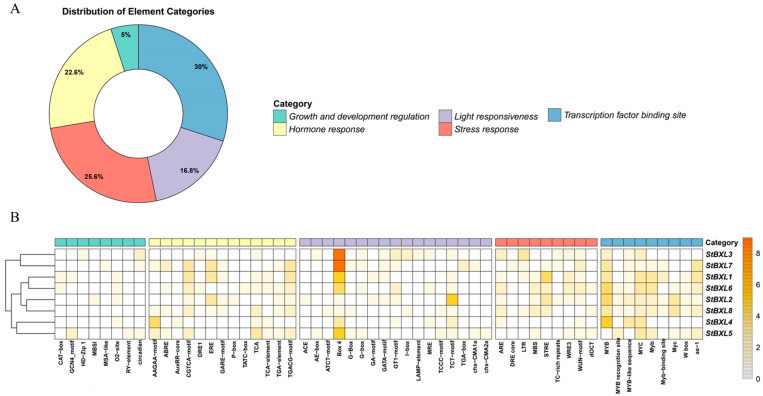
Cis-acting elements predicted in *StBXL* promoters. (**A**) Distribution of element categories in *StBXL* promoters. Green represents cis-acting elements associated with growth and development regulation, yellow represents cis-acting elements associated with hormone response, purple represents cis-acting elements associated with light responsiveness, blue represents cis-acting elements associated with transcription factor binding site, and red represents cis-acting elements associated with stress response. (**B**) Cis-acting elements predicted in *StBXL* promoters. *StBXL* promoter sequences (2 kb) were used to predict cis-acting elements on PlantCare server. The details of cis-acting elements predicted in *StBXL* promoters are listed in Table S3.

To provide insight into the regulatory mechanisms and functions of *StBXL* genes, the 2 kb upstream sequences of their translation start codons were used to scan for cis-elements. We identified five classes of cis-elements involved in the regulation of plant growth and development: light responsiveness, hormone response, stress response, and transcription factor binding sites (Figure 5A). Eight cis-elements were involved in plant growth and development regulation, thirteen cis-elements were involved in hormone responses to various signals, seventeen cis-elements were associated with light responsiveness, nine cis-elements were associated with stress responses, and nine cis-elements were binding sites of transcription factors (Figure 5B). Box 4 and MYB transcription factor binding sites were most significantly enriched in the *StBXL* promoters, suggesting that MYB transcription factor might modulate the expression of *StBXL* genes in response to stress stimuli or light signals. Several abiotic and biotic stress response elements, such as AREs, MBSs, STREs, TC-rich repeats, LTRs, and WUN-motifs, were identified in the *StBXL* promoters (Figure 5B). These findings indicated that *StBXL* genes might be involved in the potato’s responses to abiotic stresses, such as salinity, drought, and temperature, as well as to biotic stresses caused by pathogens.

### 2.6. Protein Interaction Networks of StBXL Proteins

**Figure 6:**
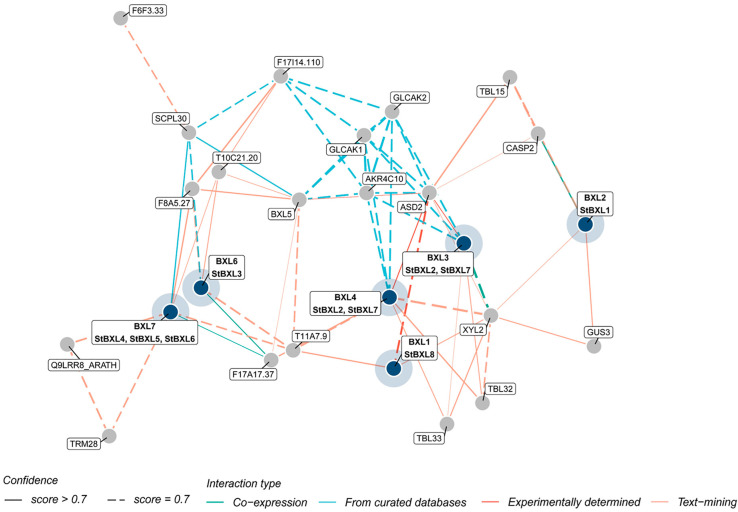
Putative protein interaction network of StBXL proteins. The homologous proteins of BXL in potato and Arabidopsis were labeled in black wireframes, with Arabidopsis BXL proteins at the top and potato proteins at the bottom. The colors of the lines indicate the different types of evidence for the prediction of the protein interaction network. The solid lines indicate a confidence score greater than 0.7; the dashed lines indicate that a confidence score was 0.7.

To investigate the potential interactions of StBXL proteins and characterize their biological functions, the STRING protein interaction database was used to integrate StBXL proteins into the Arabidopsis association model. As shown in Figure 6, the StBXL protein interaction network was inferred based on Arabidopsis homologs. StBXL1 (orthologous to AtBXL2 in Arabidopsis) was predicted to interact with XYL2, which encoded a xylosidase. StBXL8 (corresponding to AtBXL1 in Arabidopsis), StBXL2, and StBXL7 (orthologous to AtBXL3 in Arabidopsis) were predicted to have potential interactions with ASD2, which was regulated by ABA and influenced plant growth, development, and responses to various environmental stresses [41]. In addition, SCPL30 was identified as a potential interacting partner of StBXL3 (orthologous to AtBXL1 in Arabidopsis), StBXL4, StBXL5, and StBXL6 (orthologous to AtBXL7 in Arabidopsis). SCPL30 was involved in multiple key processes during plant growth and development. These processes include peptide and protein processing, post-translational modification and degradation, the regulation of plant stress resistance, growth and development, and secondary metabolite synthesis [42]. Overall, these findings indicated that StBXL proteins might have multifaceted functions.

### 2.7. Expression Patterns of StBXL Genes in Different Tissues and Under Abiotic Stresses

**Figure 7:**
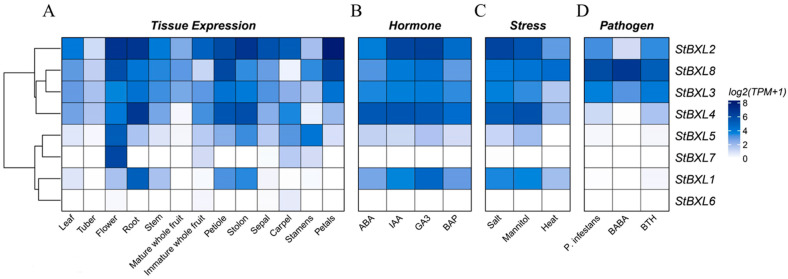
Expression profile analysis of *StBXL* genes. (**A**) Spatial transcriptome heatmap of *StBXL* genes derived from RNA-seq data (Spud DB) across 13 tissues: leaves, tubers, flowers, roots, stem, immature and mature fruits, petioles, stolons, sepals, carpels, stamens, and petals. Heat map representation of *StBXL* transcript abundance in whole potato plants after being treated with phytohormones (**B**), abiotic stress (**C**), and biotic stress (**D**). Transcript per million (TPM) values were z-score normalized and color-coded. A color gradient from white to blue represents expression levels; white represents low expression, and blue represents high expression. The dendrogram on the left side of the heatmap illustrates the hierarchical clustering of *StBXL* genes based on their expression patterns. Initial *StBXL* gene expression levels are listed in Table S4.

To investigate the potential biological functions of *StBXL* genes in potato growth and development, potato transcriptome data from the SpudDB were used to analyze *StBXL* genes’ expression in different potato tissues (Figure 7A; Table S4). All *StBXL* genes were expressed in multiple potato tissues, except *StBXL6*, which showed extremely low expression across all tissues. Roots are the first organ of the crop exposed to soil abiotic and biotic stresses. The high expression of *StBXL1*, *StBXL2*,and *StBXL4* in roots suggest their role in abiotic stress (such as salinity and alkali stress) responses in potato plants.

We also analyzed the expression patterns of *StBXL* genes under abiotic and biotic stress treatments. The analysis revealed that the expression of *StBXL4* was significantly higher than that of the other *StBXLs* under the ABA treatment, and *StBXL2, StBXL3*, *StBXL4*, and *StBXL8* exhibited higher expression levels under indole-3-acetic acid, GA_3_, and 6-benzylaminopurine (BAP) treatments, as well as under salt stress (150 mmol L^−1^ NaCl) and osmotic stress (260 mmol L^−1^ mannitol) (Figure 7B,C). However, most *StBXL* genes were insensitive to high-temperature treatments, and only *StBXL8* showed a slightly high expression. Notably, *StBXL8* exhibited the highest expression among *StBXL* genes under *phytophthora infestans* infection, DL-β-amino-N-butyric acid (BABA), and acibenzolar-S-methyl (BTH) treatments (Figure 7D), indicating its potential critical role in potato’s biotic stress response.

### 2.8. StBXL Expression Patterns Under Alkaline Stress

**Figure 8:**
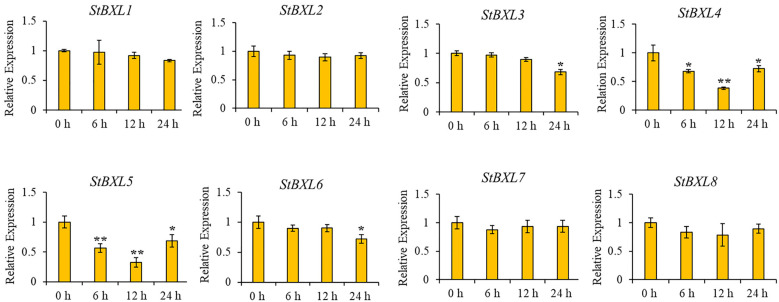
Expression profile analysis of *StBXL* genes under alkaline stress in Xisen 6. RT-qPCR analysis of *StBXL* gene expression in the root of potato cultivar Xisen 6 treated with 5 mM NaHCO_3_ for 0 h, 6 h, 12 h, and 24 h, respectively. Transcript levels were normalized to *StACT* and presented as mean ± SD (n = 3). * *p* < 0.05 and ** *p* < 0.01.

Our previous study demonstrated that the potato cultivar Xisen 6 exhibited salt stress tolerance [43]. To investigate the role of *StBXL* genes in potato’s response to alkaline stress, we analyzed the expression profiles of *StBXL* genes in Xisen 6 under an alkaline treatment using RT-qPCRs (Figure 8). The results revealed that the transcript levels of *StBXL4* and *StBXL5* were significantly downregulated under alkaline stress, with their lowest expression detected at 12 h post-treatment. However, the transcript levels of the other *StBXL* genes did not show significant differences with the alkaline treatment. These results indicated that *StBXL4* and *StBXL5* might be involved in alkaline stress responses.

### 2.9. Overexpression of StBXL4 Reduces Potato’s Resistance to Alkaline Stress

**Figure 9:**
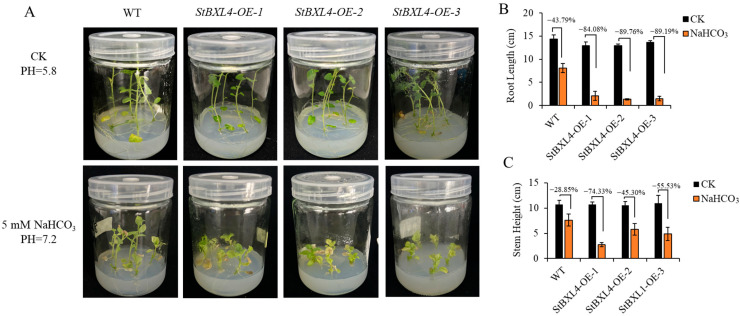
Phenotype of *OE-StBXL4* tissue culture seedlings under alkaline treatment. (**A**) The phenotype of WT and *OE-StBXL4* seedlings under CK and alkaline treatment. CK: potato stem segments with a leaf grown for 21 days in normal MS medium. Alkaline treatment: potato stem segments with a leaf grown for 21 days in MS medium containing 5 mM NaHCO_3_ (PH = 7.2). (**B**) Root length of WT and *OE-StBXL4* under alkaline treatment. (**C**) Stem height of WT and *OE-StBXL4* under alkaline treatment. Three independent lines were analyzed, and each line had ten potato seedlings for root length and stem height measurements.

To verify the functions of *StBXL4* and *StBXL5* under alkaline stress, we used the potato cultivar Xisen 6 to generate seedlings overexpressing *StBXL4* and *StBXL5*, *OE- StBXL4* and *OE-StBXL5,* respectively. The RT-qPCR analysis showed that the expression levels of *StBXL4* and *StBXL5* were higher in the transgenic lines than in the Xisen 6 (WT) (Figure S2), indicating that these transgenic lines could be used for subsequent alkaline stress experiments. Under alkaline stress, *OE-StBXL4* seedlings exhibited significantly higher root and stem growth inhibition rates compared with the WT, indicating an enhanced sensitivity to alkaline stress (Figure 9A–C). Interestingly, *StBXL5-OE* seedlings showed no significant phenotypic changes compared with the WT under alkaline stress (Figure S3). This discrepancy may be associated with the distinct subcellular localizations of *StBXL4* and *StBXL5*. These findings demonstrate that *StBXL4* negatively regulates potato’s tolerance to alkaline stress.

## 3. Discussion

The cell wall is mainly composed of cellulose, hemicellulose, and pectin [5]. Hemicellulose is mainly composed of xylan and mannan. β-D-xylosidase is a crucial enzyme in xylan hydrolysis, which synergizes with other xylanases to facilitate efficient xylan degradation [44]. Alkaline stress, a key abiotic stressor, constrains crop yield and quality. It disrupts the cellular osmotic balance, induces reactive oxygen species accumulation, and impairs roots’ cell wall structure under a high pH [35]. Potato is one of the world’s major food crops. Therefore, it is essential to explore alkali stress-related genes in potatoes.

A whole-genome analysis is fundamental for elucidating the functions and evolution of gene families. In this study, eight *StBXL* genes were identified in the potato genome, providing key targets for investigating the roles of this gene family in stress responses. A gene structure analysis revealed variations in the exon–intron distribution among these eight *StBXL* genes. However, the domains and motifs of StBXL proteins were highly conserved, reflecting the structural conservation of these proteins. These findings were consistent with those reported in other plants, such as barley [13] and soybean [14].

In an evolutionary analysis, members of the same clade typically exhibit functional similarities. StBXL1, StBXL2, StBXL4, StBXL7, and StBXL8 proteins were closely related to BXL subfamilies from Arabidopsis, soybean, and tomato, indicating that these four proteins likely perform conserved functions in these plants. However, the BXL protein functions remain poorly characterized, with only preliminary studies reported for Arabidopsis and soybean. *AtBXL1* exhibits a stem-specific expression, and its mutation leads to reduced stem and pod lengths [11]. In potatoes, the closest evolutionary homologs, *StBXL1* and *StBXL8*, were highly expressed in both flowers and stems (Figure 7), implying that they may have conserved functions in stem development, while acquiring specialized roles in floral biology. *StBXL2* and *StBXL5* were closely related to *AtBXL4* and may be involved in the metabolism of cell wall polysaccharides [16,45]. As a key rate-limiting enzyme in hemicellulose degradation, *BXL* not only plays an important role in cell wall remodeling but also participates in developmental processes. *BXL* accumulates during fruit development and plays a significant role in fruit expansion [17].

The cis-acting element analysis of the promoters revealed that the promoter regions of the eight *StBXL* genes contained abundant stress-responsive elements (e.g., MYC and MYB binding sites, MBS) and hormone-responsive elements (e.g., ARE motif and TCA motif). MYC and MYB are key transcription factors that not only regulate plant growth and development but also mediate plant responses to biotic and abiotic stresses [46,47]. These key transcription factors may bind to the promoter regions of *StBXL* genes to modulate potato growth, development, and stress responses. Additionally, the promoter region of *StBXL4* contained a large number of MBSs (Table S3), and MYC and MYB may regulate *StBXL4* expression to mediate the response to alkaline stress, though this requires further experimental validation. Serine carboxypeptidase-like proteins have been isolated from Arabidopsis [48]. *AtSCPL30* is highly expressed in roots, leaves, and flowers [49]. *TRM28* is involved in Al tolerance in Arabidopsis [50]. The protein interaction network analysis predicted that StBXL4 could interact with these proteins, suggesting that StBXL4 may regulate potato alkaline stress responses through collaboration with them. *StBXL4*, *StBXL5*, and *StBXL6* were clustered on chromosome 4 and shared conserved motifs. However, only *StBXL4* was functional under alkaline stress, which may be due to the different subcellular localizations of the three proteins, resulting in functional differentiation. A critical question for future research is to explore how *StBXL4* responds to alkali stress in potato.

In this study, we found that under alkali stress, overexpressing *StBXL4*, potato seedlings showed a hypersensitive phenotype, indicating a negative regulatory role of *StBXL4* in alkali stress. In the subcellular localization analysis, StBXL4 was localized in the extracellular, a site that is closely associated with the plant cell wall [51]. Plant cell walls are mainly composed of multiple components such as cellulose, hemicellulose, and pectin, and xylan is an important component of hemicellulose [6]. Plant cells regulate the mechanical properties and ductility of the cell wall through xylan during growth and development [52]. *StBXL4* encodes β-D-xylosidase in potato, which is one of the key enzymes for xylan hydrolysis. The alkali stress treatment inhibited the expression of the *StBXL4* gene; the low expression level of the *StBXL4* gene might reduce the degree of xylan hydrolysis under alkali stress and then inhibit cell growth, so the *OE-StBXL4* seedlings were weaker than the WT. However, the specific regulatory mechanism needs to be further explored in subsequent experiments.

## 4. Conclusions

This is the first comprehensive characterization of the potato *StBXL* gene family. Eight *StBXL* genes on five chromosomes were identified and clustered into four subfamilies based on conserved domains. A promoter analysis revealed abundant MYB/MYC binding sites, suggesting their potential regulation of *StBXL* genes. The *StBXL4* expression was significantly altered by alkali stress. Its overexpression increased potato seedlings’ alkali sensitivity, indicating a negative regulatory role in alkali responses. StBXL4 might interact with SCPL30, but the involvement of SCPL30 and the mediating role of MYB/MYC need further verification. The function of *StBXL4* in potato alkali stress responses was first validated, providing a basis for breeding alkali-tolerant varieties. Further studies are required to confirm the conservation of its function across different potato varieties.

## 5. Materials and Methods

### 5.1. Genome-Wide Identification of StBXL Genes in Potato

To identify the members of the *BXL* gene family in potatoes, potato genome data—including coding sequences, protein sequences, and genomic annotation information (GFF3 files)—were obtained from the SpudDB Potato Genomics Resource. This study retrieved seven sequences of known gene families in Arabidopsis thaliana and used TBtools-II software to perform BLASTP comparisons on the potato genome database, with the screening criterion set at E ≤ 1 × 10^−5^. The domains Glyco_hydro_3 (PF00933), Glyco_hydro_3_C (PF01915), and Fn3-like (PF14310) were extracted from the Pfam database [53] and used as queries. HMMER 3.0 software was used for comparison [54]. For multiple spliced transcripts, the longest transcript was selected for analysis. The BXL protein sequence was obtained from the NCBI Conserved Domain Database [55] and SMART website [56]. Proteins lacking conserved domains were excluded from the analysis.

### 5.2. StBXL Sequence Analysis and Characterization

ExPASy was used to quantify the amino acid count, molecular weight, and theoretical pI of the StBXL proteins. The subcellular localization of the proteins was predicted using WoLF PSORT II [57]. The MEME online tool was used to analyze conserved motifs of the StBXL proteins, with a maximum of 10 motifs and an optimal motif width of six to fifty amino acid residues. The gene structure viewer and gene location visualization functions in TBtools were used to analyze and display the structures and chromosomal locations of the *StBXL* genes [58].

### 5.3. Prediction of Cis-Elements and Protein Interaction Networks of StBXL

The 2 kb upstream sequences of the StBXL genes were extracted from the potato genome. The PlantCARE database was used to screen for potential cis-elements in the promoters of *StBXL* genes [59]. Systematic construction of possible interactions was performed using the protein–protein interaction prediction method. Potato BXL family members were mapped to Arabidopsis BXL family members, and a high confidence threshold (confidence score ≥ 0.7) was set to ensure the reliability of the interactions. Multiple sources of information—including predicted interactions, experimental validation, interolog mapping, and literature mining—were integrated.

### 5.4. Phylogenetic Tree Construction and Gene Duplication and Synteny Analysis

To investigate whether StBXL proteins have different evolutionary relationships in monocotyledons and dicotyledons, we selected dicotyledons (Arabidopsis, rice, soybean, and tomato) and monocotyledons (maize) for phylogenetic tree construction and representatives of the lycopersicon family (tomato, pepper, and tobacco) for synteny analysis. The URLs for the genomic data of the species used to construct the phylogenetic tree were as follows:

Arabidopsis (https://www.arabidopsis.org/, accessed on 20 May 2025);
Tomato (https://solgenomics.net/, accessed on 20 May 2025);
Maize (https://plants.ensembl.org/Zea_mays/Info/Index, accessed on 20 May 2025);
Soybean (https://plants.ensembl.org/Glycine_max/Info/Index, accessed on 20 May 2025);
Rice (https://rice.uga.edu/pub/data/Eukaryotic_Projects/o_sativa/, accessed on 20 May 2025);
Pepper and tobacco (http://plants.ensembl.org/info/data/ftp/index.html, accessed on 20 May 2025).

The amino acid sequences of the *StBXL* genes were saved in FASTA format, and multiple sequence alignments of the StBXL proteins were performed using the MAFFT program with default parameters. A phylogenetic tree was constructed using IQ-TREE 3.0.1 software (https://iqtree.github.io/, accessed on 20 May 2025) with the maximum likelihood method, setting the bootstrap value to 1000. The iTOL tool was used to beautify the phylogenetic tree. MCScanX was used for gene duplication and collinearity analyses, and TBtools was used for visualization. Ka/Ks Calculator (v.2.0) was used to calculate synonymous (Ks) and nonsynonymous (Ka) substitution rates.

### 5.5. RNA-Seq Data Source and Expression Pattern Analysis of StBXL Genes

To explore the expression patterns of *StBXL* genes, RNA-seq expression profiles from SpudDB were used to analyze *StBXL* genes’ expression in various tissues of doubled monoploid potatoes [40]. Heatmaps of *StBXL* genes expression levels were visualized using the Heatmap Illustrator program in the TBtools toolkit.

### 5.6. Genetic Transformation of Potato and Identification of Transgenic Plants

The coding sequences of the *StBXL1* and *StBXL5* genes were amplified according to the designed specific primers (Table S5) and inserted into the *pK7FWG2* vector. Agrobacterium suspensions containing the recombinant plasmids were cultured in LB liquid medium (containing 50 µg/mL rifampicin and 50 µg/mL spectacularin) and activated to OD600 = 0.5. They were then transformed into Xisen 6 by the Agrobacterium-mediated stem segmentation method. *StBXL1* and *StBXL5* transgenic overexpression lines were obtained by antibiotic screening and PCR confirmation. Fifteen individual transgenic lines were obtained and further verified by spectacularin and qRT-PCR. Among these transgenic potato lines, three independent transgenic lines were selected and grown in an incubator and used for subsequent experiments. Growth conditions were 23 °C, 16 h light/8 h dark photoperiod, and a light intensity of 10,000 Lx.

### 5.7. Alkali Stress Treatment and Phenotypic Identification of Potato Seedlings

Under alkaline stress, the potato cultivar Xisen 6 was used for the expression analysis of *StBXL* genes. Tissue culture seedlings that grew vigorously in medium were selected, and stem segments were trimmed. The trimmed stem segments containing young leaves were placed on solid MS medium for 21 d. Seedlings with similar growth were selected and pre-cultured in MS broth for 1 d. The seedlings were then transferred to an alkali medium containing 5 mmol L^−1^ NaHCO_3_ (pH 7.2) and treated for different durations (0 h, 6 h, 12 h, and 24 h). Roots were collected at each time point and quickly frozen in liquid nitrogen for RNA extraction. Reverse transcription was performed using a reverse transcription kit, and the cDNA was used as a template for qRT-PCR.

WT and *OE-StBXL4* transgenic potato seedlings were cultured in MS medium for 21 d. Well-growing WT and transgenic tissue culture seedlings were selected for stem segment trimming. Trimmed stem segments contained one leaf each. Segments with consistent growth were cultured separately on MS solid medium or alkaline solid medium (5 mmol L^−1^ NaHCO_3_, pH 7.2). After 21 d of growth, the seedlings were removed from the medium, and the relevant indicators were measured.

### 5.8. Data Analysis

SPSS Statistics 26 was used for significance analysis. Differences between samples were considered significant at *p* < 0.05. Microsoft Excel 2016 was used for data organization and preliminary analysis.

## Figures and Tables

**Table 1:** Physicochemical properties of *StBXL* family proteins in potato.

Gene Name	Locus ID	Chromosome Location	Number of Amino Acids (aa)	Protein MW (KDa)	Theoretical pI	Instability Index	Subcellular Localization
*StBXL1*	Soltu.DM.01G024730.1	Chr01	774	83,826.65	8.67	37.00	Chloroplast
*StBXL* *2*	Soltu.DM.01G044180.1	Chr01	778	85,236.29	8.50	28.24	Endoplasmic Reticulum
*StBXL* *3*	Soltu.DM.02G026960.1	Chr02	793	87,360.26	6.10	35.34	Plasma Membrane
*StBXL* *4*	Soltu.DM.04G029040.1	Chr04	775	85,171.01	8.01	30.54	Extracellular
*StBXL* *5*	Soltu.DM.04G029050.1	Chr04	774	85,184.09	6.98	31.39	Chloroplast
*StBXL* *6*	Soltu.DM.04G029120.1	Chr04	779	86,171.92	9.09	29.82	Plasma Membrane
*StBXL* *7*	Soltu.DM.10G012310.1	Chr10	836	91,199.12	8.26	28.46	Vacuole
*StBXL* *8*	Soltu.DM.11G017090.1	Chr11	773	83,904.52	7.32	39.42	Chloroplast

## Data Availability

Data from this study are available in the article and Supplementary Materials.

## Supplementary Materials

The following supporting information can be downloaded at https://www.mdpi.com/article/10.3390/plants14243790/s1: Figure S1. Motif sequence identified for StBXL proteins; Figure S2. Expression profile analysis of StBXL4 and StBXL5 under alkaline treatment in overexpressing plant; Figure S3. Root length and stem height of WT and OE-StBXL5 under alkaline treatment; Table S1. StBXL sequence data; Table S2. The value of Ka/Ks in synteny analysis; Table S3. Analysis results of cis-acting components; Table S4. Transcriptomic data for StBXL gene expression patterns in different tissues and different treatments; and Table S5. Primers used in this experiment.
