# Supplementary material for: Genome-Wide Identification of β-D-Xylosidase Gene Family in Potato and Functional Analysis Under Alkaline Stress
Source: Plants (Basel). 2025 Dec 12;14(24):3790. doi: 10.3390/plants14243790 (PMC12736900; doi:10.3390/plants14243790)
Supplement: Supplementary file 1 [file plants-14-03790-s001.zip › Figure S3. Root length and stem height of WT and OE-StBXL5 under alkaline treatment.pdf]

Figure S3. Root length and stem height of WT and *OE-StBXL5* under alkaline treatment

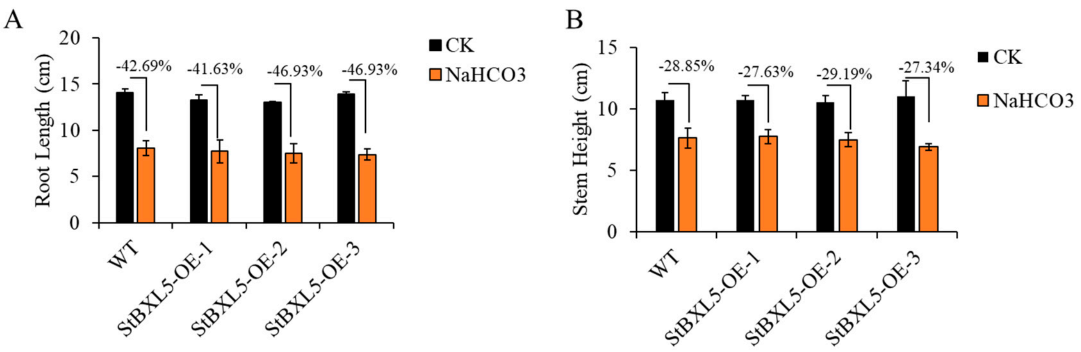

Root length of WT and *OE-StBXL5* under CK and alkaline treatment(A). Stem height of WT and *OE-StBXL5* under CK and alkaline treatment(B).
