# Supplementary figures and images for: Genome-Wide Identification of β-D-Xylosidase Gene Family in Potato and Functional Analysis Under Alkaline Stress
Source: Plants (Basel). 2025 Dec 12;14(24):3790. doi: 10.3390/plants14243790 (PMC12736900; doi:10.3390/plants14243790)

Figure S1. Motif Sequence identified for StBXL proteins

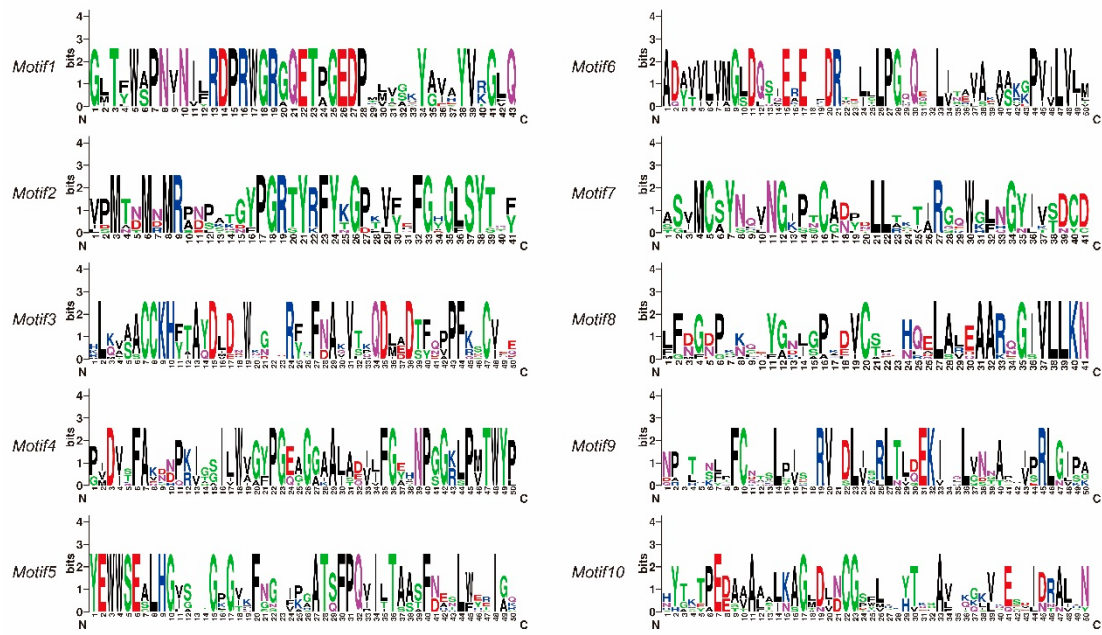

Supplement: Supplementary file 1 [file plants-14-03790-s001.zip › Figure S1. Motif Sequence identified for StBXL proteins.pdf]

Figure S2. Expression profile analysis of *StBXL4* and *StBXL5* under alkaline in overpression plant.

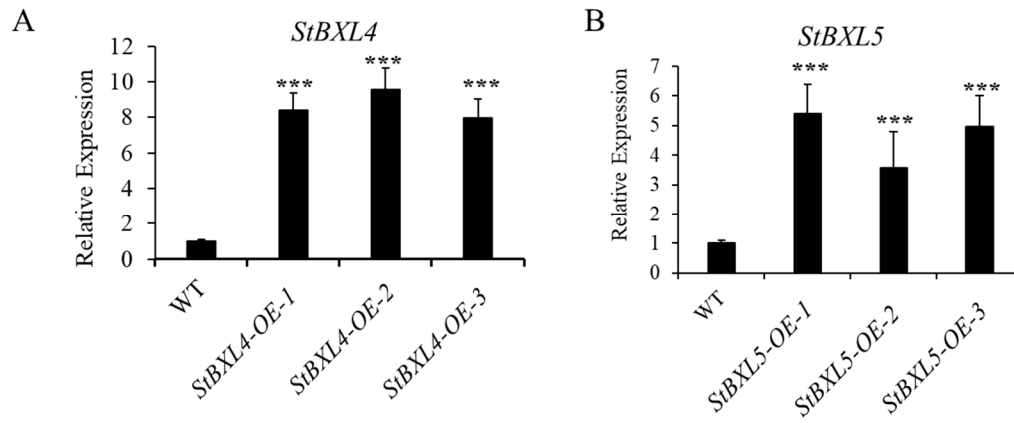

Supplement: Supplementary file 1 [file plants-14-03790-s001.zip › Figure S2.Expression profile analysis of StBXL4 and StBXL5 under alkaline in overpression plant.pdf]
